# Improved Melt Processabilities of Thermosetting Polyimide Matrix Resins for High Temperature Carbon Fiber Composite Applications

**DOI:** 10.3390/polym14050965

**Published:** 2022-02-28

**Authors:** Hao-Yang Zhang, Li-Li Yuan, Wei-Jie Hong, Shi-Yong Yang

**Affiliations:** 1 Key Laboratory of Science and Technology on High-Tech Polymer Materials, Institute of Chemistry, Chinese Academy of Sciences, Beijing 100190, China; zhanghy@iccas.ac.cn (H.-Y.Z.); hongweijie@iccas.ac.cn (W.-J.H.); 2 School of Chemical Sciences, University of Chinese Academy of Sciences, Beijing 100190, China

**Keywords:** phenylethynyl-endcapped poly(amic ester), melt processability, mechanical properties, thermal properties

## Abstract

With the goal of improving processability of imide oligomers and achieving of high temperature carbon fiber composite, a series of Thermosetting Matrix Resin solutions (TMR) were prepared by polycondensation of aromatic diamine (3,4′-oxybisbenzenamine, 3,4-ODA) and diester of biphenylene diacid (BPDE) using monoester of 4-phenylethynylphthalic acid (PEPE) as end-capping agent in ethyl alcohol as solvent to afford phenylethynyl-endcapped poly(amic ester) resins with calculated molecular weight (Calc’d M_w_) of 1500–10,000. Meanwhile, a series of reactive diluent solutions (RDm) with Calc’d M_w_ of 600–2100 were also prepared derived from aromatic diamine (4,4′-oxybisbenzenamine, 4,4-ODA), diester of asymmetrical biphenylene diacid (α-BPDE) and monoester of 4-phenylethynylphthalic acid (PEPE) in ethyl alcohol. Then, the TMR solution was mixed with the RDm solution at different weight ratios to afford a series of A-staged thermosetting blend resin (TMR/RDm) solutions for carbon fiber composites. Experimental results demonstrated that the thermosetting blend resins exhibited improved melt processability and excellent thermal stability. After being thermally treated at 200 °C/1 h, the B-staged TMR/RDm showed very low melt viscosities and wider processing window. The minimum melt viscosities of ≤50 Pa·s was measured at ≤368 °C and the temperature scale at melt viscosities of ≤100 Pa·s were detected at 310–390 °C, respectively. The thermally cured neat resins at 380 °C/2 h showed a great combination of mechanical and thermal properties, including tensile strength of 84.0 MPa, elongation at breakage of 4.1%, and glass transition temperature (T_g_) of 423 °C, successively. The carbon fiber reinforced polyimide composite processed by autoclave technique exhibited excellent mechanical properties both at room temperature and 370 °C. This study paved the way for the development of high-temperature resistant carbon fiber resin composites for use in complicated aeronautical structures.

## 1. Introduction

Aromatic polyimide materials such as films, fibers, engineering plastic, matrix resins and coatings, due to their excellent combined thermal, mechanical and electrical properties [1,2], have been extensively used in many high-tech industries such as aerospace and aviation [3,4,5], microelectronics [6,7,8] and opto-electric display [9,10,11], etc. Aromatic polyimide could be divided into thermosetting and thermoplastic resins, of which the thermosetting polyimide resins have been extensively employed to produce carbon (or quartz) fiber reinforced polyimide composite components for 300–450 °C applications in aeronautics and astronautics [12,13,14]. However, due to the enhanced backbone rigidity and polymer chain length, the melt processability of the thermosetting polyimide resins are deteriorated obviously with increasing the long-term servicing temperature [15,16,17].

The first generation of high temperature resistant polyimide resin represented by PMR-15 can be used at 316 °C for a long time, and its carbon fiber composites have excellent mechanical properties, while the melt viscosity is lower than 100 Pa·s, which is suitable for hot press tank process [18,19]. The second generation of high temperature resistant polyimide resin represented by PMR-II-50 adopts 6FDA monomer containing -CF_3_ structure, and the long-term use temperature reaches 371 °C, but the resin has high melt viscosity, poor flowability, and easy to prepare composites with high porosity [20,21]. For this reason, mostly used in hot molding to prepare bearings and bushings for aircraft, etc. It is difficult to prepare structural parts with complex structures. In recent years, significant efforts have been devoted to improving the melt processability of thermosetting polyimide resins by introducing soft segments into polyimide backbone [22,23], or by reducing polymer molecular weight [24], or blending reactive diluents with polyimide resin to produce interpenetrating network [25,26]. Essentially, end-cap chemistry and polymerization structure play a key role in controlling the flow of the oligomer and the stability of the curing resin [27,28,29], but a highly cross-linked chemical structure can compromise the strength and toughness of the polyimide resins [30,31]. Therefore, it is difficult to combine processing and heat resistance properties by adjusting the resin backbone alone. It was found that blending reactive diluent with thermosetting polyimide resins was an effective pathway to improve the resin melt processability due to the disturbing polymer chain packing density and the debilitating intermolecular interaction [32].

Rao et al. have synthesized a tribranched phenylethynyl-terminated aryl ether compound (Tri-PE-PAEK), which was used as a reactive diluent for phenylethynyl-terminated imide oligomer (PETI-5) with Calc’d M_w_ of 5000 g/mol. It was found that the minimum melt viscosity of the blended resin (PETI-5/Tri-PE-PAEK) was reduced significantly by increasing the Tri-PE-PAEK weight loading level. At 30 wt.% of the Tri-PE-PAEK loading level, the minimum melt viscosity of the blended resin decreased to 40% of the intrinsic viscosity of PETI-5 [33]. Wu et al. have synthesized a carborane-containing monofunctional imide monomer (CB-PEPA), which was used as a reactive diluent for phenylethynyl-terminated imide oligomer (PETI) to reduce the minimum melt viscosity and broaden the process window of composite. The composite derived from 10% of CB-PEPA weight loading in PETI/carbon fiber composite exhibited a relatively higher flexural strength and interlaminar shear strength [34]. Yu et al. have synthesized a reactive diluent (ODA-PEPA) and a flexible phenylethynyl-terminated imide oligomer (PEI-PEPA). The ODA-PEPA/PEI-PEPA blend resins showed lower melt viscosity and wider processing window, and the melt processabilities of the advanced composites were apparently improved [26].

In our previous report, a reactive dilute derived from 4-phenylethynyl phthalic anhydride (PEPA) and meta-phenylenediamine (m-PDA) have been synthesized, which showed effective improvement in melt processing performance of the thermosetting polyimide matrix resins for 350 °C applications [35]. In this work, a series of phenylethynyl-endcapped oligomers with Calc’d M_w_ of 600–2100 were prepared, which were used to dilute the thermosetting polyimide matrix resins for 370 °C carbon fiber composite applications. The influence of Calc’d M_w_ of the reactive diluents as well as thermosetting poly(amic ester) resins on the melt processability, thermal and mechanical properties of the thermosetting blend resins have been systematically investigated.

## 2. Materials and Methods

### 2.1. Materials

3,3′,4,4′-Biphenyl tetracarboxylic dianhydride (BPDA, >99%) and 2,3′,3,4′-biphenyl tetracarboxylic dianhydride (α-BPDA, >99%) were purchased from JFE Chemical Corporation, and purified by vacuum dehydration at 110 °C for 12 h prior to use. 3,4′-oxybisbenzenamine (3,4-ODA, >99%), 4,4′-oxybisbenzenamine (4,4-ODA, >99%) and 4-phenylethynylphthalicanhydride (PEPA, >99%) were supplied by Changzhou Sunlight Pharmaceutical Co. Ltd., Changzhou, China and used as received. Commercially available anhydrous ethanol obtained from Chemical Reagent Beijing Co. Ltd. (Beijing, China) was stored with 4 Å molecular sieves prior to use.

### 2.2. Polymer Synthesis

#### 2.2.1. Synthesis of Thermosetting Poly(amic ester) Resins (TMR)

In a 100 mL three-necked, round-bottomed flask equipped with a nitrogen inlet, a condenser and a Teflon-coated magnetic stirring bar, BPDA (27.8592 g, 0.0947 mol) was refluxed for 4 h in anhydrous ethanol (41.1559 g, 52.2 mL) to give diethyl ester of 3,3′,4,4′-biphenyl dicarboxylic acid (BPDE). Then, PEPA (4.9646 g, 0.0200 mol) and anhydrous ethanol (18.9360 g, 24.0 mL) were added and refluxed for 4 h to afford 4-phenylethynylphthalic acid monoester (PEPE). Finally, 3,4-ODA (20.9481 g, 0.1047 mol) and anhydrous ethanol (5.0496 g, 6.4 mL) were added and stirred for 18 h to afford A-staged thermosetting matrix resin solution with Calc’d M_w_ of 5000 and solid content of 50% (TMR–50).

Similarly, TMRs with different Calc’d M_w_ of 1500–10,000, including TMR-15 (Calc’d M_w_ =1500), TMR-25 (Calc’d M_w_ = 2500), TMR-35 (Calc’d M_w_ = 3500), TMR-75 (Calc’d M_w_ = 7500), TMR-100 (Calc’d M_w_ = 10,000), were prepared derived from BPDE, 3,4-ODA and PEPE, respectively.

#### 2.2.2. Synthesis of Reactive Diluents (RDm)

In a typical experiment, RDm–b with Calc’d M_w_ of 1119 g/mol was prepared in the following procedure. In a 100 mL three-necked flask, a-BPDA (13.1487 g, 0.0447 mol) and anhydrous ethanol (21.1559 g, 26.8 mL) were refluxed for 4 h to yield diethyl ester of 2,3,3′,4′-biphenyl dicarboxylic acid (a-BPDE) solution. Meanwhile, PEPA (22.1868 g, 0.0900 mol) and ethanol (28.6790 g, 36.4 mL) were refluxed in a 100 mL flask for 4 h to afford monoester of 4-phenylethynylphthalic acid (PEPE). After *a*-BPDE solution and PEPE solution was mixed to give a homogeneous solution, ethanol (17.3580 g, 22.0 mL) and 4,4-ODA (17.8849 g, 0.0894 mol) were then added with stirring. The suspension was stirred in nitrogen at room temperature overnight to give a phenylethynyl-endcapped oligomer solution with a solid content of 50 wt.% and Brookfield viscosity of 34 mPa·s (RDm–b).

Similarly, RDm–c with Calc’d M_w_ of 1577 and RDm–d with Calc’d M_w_ of 2035 were also prepared derived from PEPE, *a*-BPDE, 4,4-ODA, respectively. Additionally, RDm–a with Calc’d M_w_ of 661 was prepared by reaction of PEPE and 4,4-ODA at a mole ratio of 2:1.

#### 2.2.3. Synthesis of Thermosetting Blend Resins (TMR/RDm)

In a typical experiment, 100 g of TMR–50 solution with a solid content of 50% was placed into a 250 mL three-necked, round-bottomed flask equipped with a nitrogen inlet, and a Teflon-coated magnetic stirring bar. Then, 50 g of RDm–a solution with a solid content of 50% was mixed with the RDm–a solution with stirring at room temperature to afford an A-staged homogeneous blend matrix resin solution (TMR–50/RDm–a–5) with a solid content of 50% and Brookfield viscosity of 30 MPa·s at room temperature.

The as-prepared A-staged blended resin solution was evaporated with a rotary evaporator at 60 °C/1 h and 120 °C/1 h to remove most of the ethanol and cumulatively heated at 150 °C/1 h, 200 °C/1 h for thermal treating to afford the B-staged TMR–50/RDm–a–5 blend resins.

Similarly, TMR–50/RDm–b–5 derived from TMR–50 and RDm–b at a weight ratio of 50/50, TMR–50/RDm–c–5 derived from TMR–50 and RDm–c at a weight ratio of 50/50, TMR–50/RDm–d–5 derived from TMR–50 and RDm–d at a weight ratio of 50/50, were prepared, respectively. And TMR-15/RDm–a–5 derived from TMR-15 and RDm–a at a weight ratio of 50/50, TMR-25/RDm–a–5 derived from TMR-25 and RDm–a at a weight ratio of 50/50, TMR-35/RDm–a–5 derived from TMR-35 and RDm–a at a weight ratio of 50/50, TMR-75/RDm–a–5 derived from TMR-75 and RDm–a at a weight ratio of 50/50, TMR-100/RDm–a–5 derived from TMR-100 and RDm–a at a weight ratio of 50/50, were also prepared (Table 1).

#### 2.2.4. Thermally Cured Blend Neat Resins

**Scheme 1:**
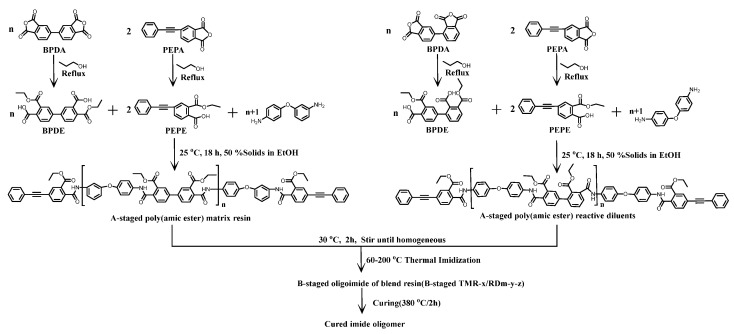
Preparation process of cured resin blends.

In a typical experiment, the B-staged TMR–50/RDm–a–5 resin powder was placed in stainless steel die, then thermally processed at 300 °C/0.5 h under a contacted pressure in a Heated Laboratory Press (IDM Instruments Pty Ltd., Australia). A pressure of 1.5 MPa was exerted when the temperature was ramped up to 350 °C. Then, the temperature increased from 350 °C to 380 °C at a rate of 5 °C/min. and the applied pressure increased to 2.0 MPa to avoid undesirable voids or defects induced by volatiles. After standing at 380 °C for 2 h, the die was cooled down to 100 °C and the applied pressure was released. The thermally cured blend neat resin plate was obtained (Scheme 1).

#### 2.2.5. Preparation of Carbon Fiber Composites

**Figure 1:**
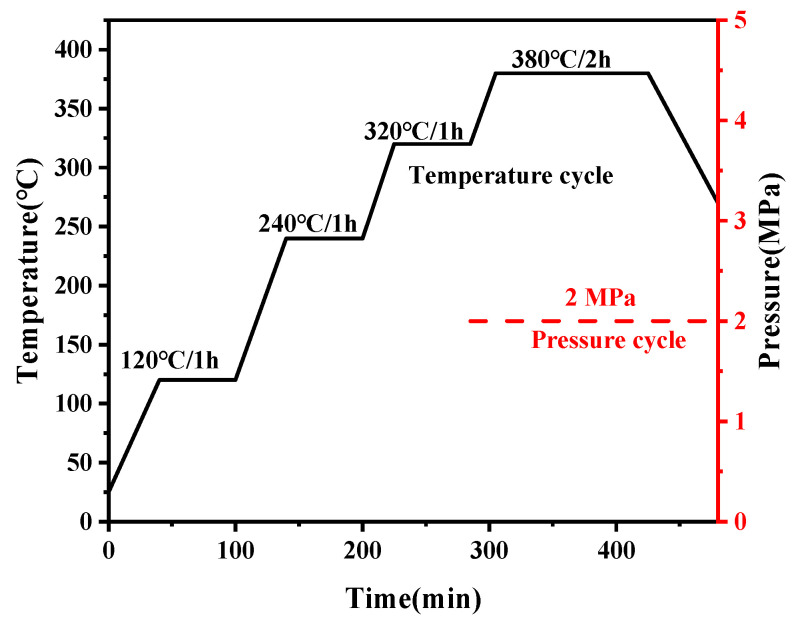
Processing cycle of the polyimide/carbon fiber composite.

Prepreg was prepared by first coating the surface of T700 carbon fiber using A-staged blend resin solution (TMR–50/RDm–a–5), followed by drying at 30 °C for 24 h, in which the organic volatile contents of as-prepared prepregs was controlled in the range of 10–15 wt.%. A small fraction of volatiles that remained in the prepreg was employed to prevent the delamination of prepregs during the composite processing. The polyimide/carbon fiber composites were fabricated from the stacked prepregs by autoclave process at high temperature. Figure 1 depicts the processing cycle of polyimide/carbon fiber composite.

The low boiling point volatiles was promptly expelled by heating the prepregs at 120 °C/1 h, then 240 °C/1 h and 320 °C/1 h at a contacted pressure. The temperature was then ramped to 380 °C with a heating rate of 3 °C/min at an applied pressure of 2 MPa, and then held at 380 °C/2 h. The applied pressure was kept after the temperature was cooled down to 250 °C. After cooled down to room temperature, the thermally cured polyimide/carbon fiber composite laminate was then removed from the mold, and the mechanical properties were evaluated at room temperature and 370 °C, respectively.

### 2.3. Characterization and Measurements

Melt rheological behavior of B-staged imide oligomer was performed using an AR2000 rheometer (TA Instruments, New Castle, DE, USA) equipped with 25-mm-diameter parallel plate fixture measured at the heating rate of 4 °C/min. The upper plate was oscillated at a fixed strain of 0.1% and a constant angular frequency of 10 rad/s, while the lower one was attached to a transducer that recorded the resultant torque and then converted to the complex viscosity. A TA Q100 thermal analysis system under nitrogen purge at a heating rate of 10 °C/min was used to perform differential scanning calorimetry (DSC). Dynamic mechanical analysis (DMA) was executed on a TA Q800 analyzer with a heating rate of 5 °C/min and a load frequency of 1 Hz in nitrogen atmosphere ranging from 40 to 550 °C. Three-point bending mode was applied with the specimen size of 20 mm × 5 mm × 2 mm, and the T_g_ was taken at the inflection temperature in the storage modulus (E′) versus temperature curve. Thermogravimetric analysis (TGA) was performed on a TA Q50 instrument at a heating rate of 10 °C/min under a 40 mL/min flowing air and nitrogen purge. Thermal mechanical analysis (TMA) was performed on a TA Q400 instrument at a heating rate of 10 °C/min under a 40 mL/min flowing nitrogen purge.

Mechanical characteristics of the thermally-cured IPNs were determined at 25 °C via an Instron 5567 universal testing machine (Instron, Boston, MA, USA), and the resultant values represented the average of at least seven runs per specimen. Tensile strength and modulus of 5.0-mm wide IPN sample were acquired at a crosshead speed of 1.0 mm/min following GB/T 1040–2006, and flexural strength and modulus of 3.0-mm-wide one were obtained at a rate of 1.0 mm/min in accordance with HG/T 3840–2006, respectively.

## 3. Results

### 3.1. Characterization of the Thermosetting Blend Resins

**Figure 2:**
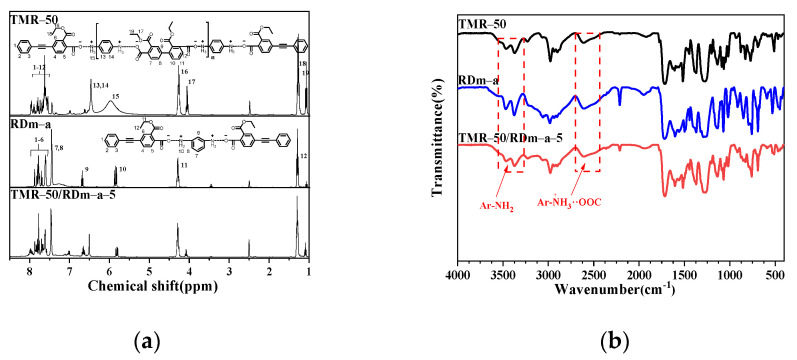
^1^H NMR (**a**) and FT-IR spectra (**b**) of A-staged blend resin (TMR–50/RDm–a–5) and that of thermosetting matric resin (TMR–50) and reactive diluent (RDm–a).

Figure 2a compares ^1^H-NMR of the thermosetting blend resin (TMR–50/RDm–a–5) with the thermosetting matrix resin (TMR–50) and the reactive diluent (RDm–a). The peaks at 4.0–4.2 ppm (H16 and H17) and 1.05–1.25 ppm (H18 and H19) were designed as the protons of the ester groups in TMR–50. The broad peak located at 6.0 ppm (H15) was attributed to the protons of the amide (=N-H); The absorptions at 6.5 ppm (H10 to H14) were designed as the protons of phenyl rings to which the amine is attached, and others designed as the protons of the aromatic rings [36]. The ^1^H NMR spectrum of RDm–a also indicated that it has the expected chemical structure, in which the peak at 4.2 ppm (H10) and that at 1.25 ppm (H11) were designed as the protons of the ester groups of PEPE. The thermosetting blend resin (TMR–50/RDm–a–5) derived from the thermosetting matrix resin (TMR–50) and the reactive diluent (RDm–a) at a weight ratio of 50:50 showed an exactly combinatorial ^1^H NMR spectra of TMR–50 and RDm–a.

Figure 2b compares FT-IR spectra of the thermosetting blend resin (TMR–50/RDm–a–5) with the thermosetting matrix resin (TMR–50) and the reactive diluent (RDm–a). The characteristic absorptions around 3458 cm^−1^ and 3365 cm^−1^ correspond to the stretching vibrations of –N-H in aromatic primary amines, and the medium intensity absorption at 2500 cm^−1^ is the characteristic absorption of the primary amine salt [37]. In addition, neither of them detected the signature -C=O asymmetric stretching vibration absorption peak of the imine ring at 1780 cm^−1^, but the broad peaks of -C=O and -C-N were detected at 1720 cm^−1^ and 1360 cm^−1^, respectively, indicating that no imide ring was generated and the system was mainly dominated by amides obtained by the reaction of diamine and tetracarboxylic diester [38]. However, the characteristic absorptions around 2210 cm^−1^ attributed to the stretching vibrations of -C≡C- in phenylethynyl groups were observed in both TMR–50/RDm–a–5 and RDm–a, which was not detected in TMR–50 due to its low contents of phenylethynyl groups.

**Figure 3:**
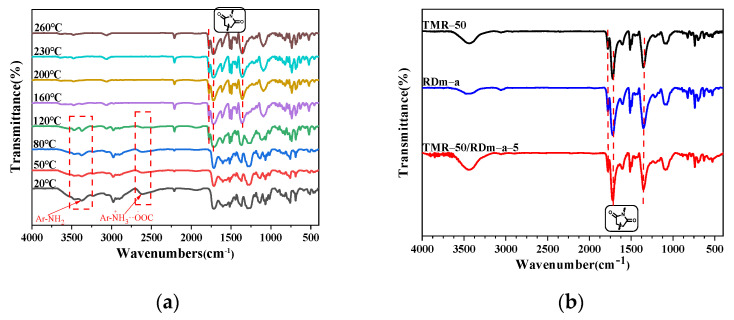
FT-IR spectra of the thermosetting blend resin (TMR–50/RDm–a–55) were cured at different temperatures (20–260 °C) (**a**) and cured at 380 °C/2 h (**b**).

Figure 3a depicts FT-IR spectra of the thermosetting blend resin (TMR–50/RDm–a–5) cured at different temperatures (20–260 °C). When the treatment temperature was increased to ≥120 °C, the characteristic absorptions of the polyester amine salt in the range of 2500–2800 cm^−1^ and 3250–3500 cm^−1^ were disappeared completely. And the characteristic absorptions of the imide ring structure at 1780, 1720 and 1370, 740 cm^−1^ appeared, due to the C=O asymmetric and symmetric stretching vibrations and the C-N stretching and bending vibrations, and their intensities were increased with the increasing treatment temperature of the blend resins. In addition, the absorptions at 2210 cm^−1^, attributed to the carbon-carbon stretching vibrations in phenylethynyl groups (-C≡C-), were not disappeared even after being thermally treated at 260 °C, implying that no obvious thermal curing reaction occurred [39]. The characteristic peaks of isoimide around 1800 cm^−1^ and 905 cm^−1^ were not detected, implying that the imidization process didn’t produce the corresponding isoimide groups [40]. Figure 3b compares the FT-IR spectra of the thermally cured blend resin (TMR–50/RDm–a–5) with the thermosetting matrix resin (TMR–50) and the reactive diluent (RDm–a) after cured at 380 °C/2 h. Obviously, the thermally cured TMR–50/RDm–a–5 exhibited the expected chemical structures.

### 3.2. Melt Processability of the Thermosetting Blend Resins

**Figure 4:**
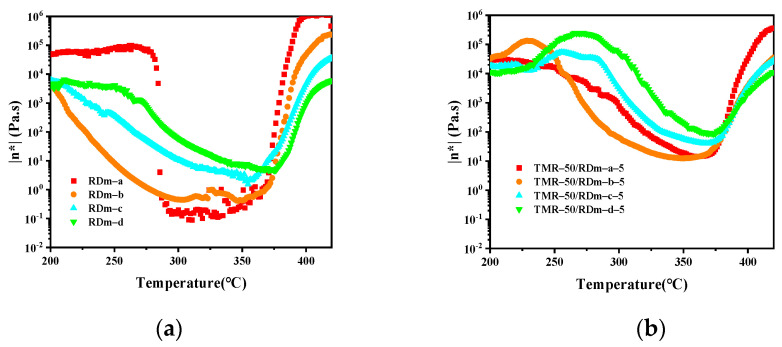
Dynamic rheological properties of the B-staged reactive diluents with different Calc’d M_w_ (**a**) and the B-staged blend resins (TMR–50/RDm–*x*–5) at a weight ratio of 50/50 (**b**).

The reactive diluents (RDm) were prepared by polycondensation of *a*-BPDE, 4,4-ODA and PEPE at Calc’d M_w_ of 660 (RDM–a, n = 0), 1120 (RDm–b, n = 1), 1580 (RDm–c, n = 2) and 2040 (RDm–d, n = 3), respectively. The minimum melt viscosities (|η*|_min_) of the RDm increased gradually with the rising of the Calc’d M_w_ of RDm (Figure 4a). For instance, RDm–b showed a |η*|_min_ of 3.81 Pa·s at 355 °C, lower than that of RDm–c (39.8 Pa·s at 368 °C) and RDm–d (86.1 Pa·s at 373 °C), respectively. Moreover, RDm–b exhibited the temperature scale at 10 Pa·s (Δ*T*_10_) was 250–375 °C, much lower than RDm–c (Δ*T*_10_ = 300–375 °C) and RDm–d (Δ*T*_10_ = 340–380 °C), respectively (Table 2). The RDm was used to melt dilute the thermosetting matrix resin (TMR–50) at a weight ratio of 50/50 to afford a series of the thermosetting blend resins, including TMR–50/RDm–a, TMR–50/RDm–b, TMR–50/RDm–c and TMR–50/RDm–d, respectively. Compared to the un-diluted resin TMR–50, the B-staged blend resins thermally treated at 200 °C /1 h showed obviously reduced melt viscosities (Figure 4b), in which TMR–50/RDm–b has the lowest melt viscosity of 3.8 Pa·s at 358 °C and TMR–50/RDm–d has the highest melt viscosity of 86.1 Pa·s at 360 °C, respectively. The melt processing temperature window defined as the temperature scale at melt viscosity of lower than 100 Pa·s was measured in the range of 293–380 °C, in which TMR–50/RDm–b showed the broad processing window (293–380 °C), better than TMR–50/RDm–c (335–380 °C) and TMR–50/RDm–d (363–380 °C), respectively (Table 2).

**Figure 5:**
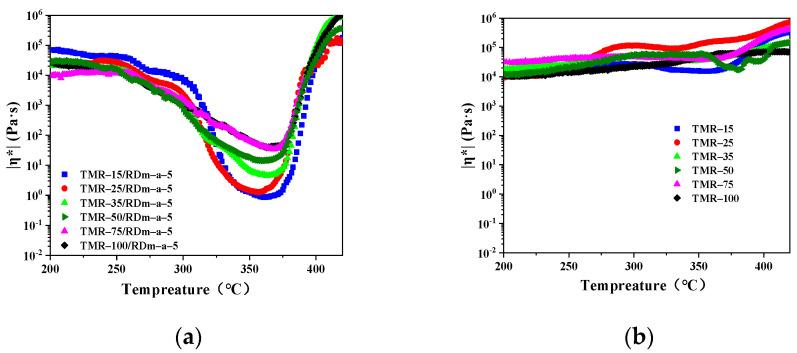
Melt viscosity of the B-staged TMR (**a**) and the blended resin TMR/RDm (50/50) (**b**) at different temperatures (200–400 °C).

Additionally, the molecular weight of the matrix resin also showed apparent impacts in the melt processing properties of the blend resins. Figure 5 compares the dynamic rheological properties of the B-staged thermosetting matrix resins with different Calc’d M_w_ of 1500–10,000 (TMRs) and the B-staged blend resins (TMR/RDm–a). The B-staged TMRs showed melt viscosities of higher than 1.0 × 10^4^ Pa·s at a temperature of 200–400 °C (Figure 5a), implying that the thermosetting matrix resins could not be melted processed by the autoclave technique. For instance, the B-staged thermosetting matrix resin with Calc’d M_w_ of 5000 (TMR–50) showed a minimum melt viscosity of 2.44 × 10^4^ Pa·s at 370 °C, showing very poor melt processability.

After mixing the reactive diluent (RDm–a) with the matrix resins (TMR) with different Calc’d M_w_ of 1500–10,000 g/mol at weight ratios of 50/50, all of the blended resins (TMR/RDm–a–5) showed significant improvement in melt processabilities. With the Calc’d M_w_ of TMR changed from 1500 to 10,000 g/mol, the minimum melt viscosity of the blended resins increased from 1.7 Pa·s to 40.6 Pa·s, and the temperature scales at 100 Pa·s (Δ*T*_100_) was measured at 317–388 °C (Table 3), demonstrating that diluent blending has a significant viscosity reduction effect on high molecular weight matrix resins. For instance, TMR–50/RDm–a–5 has the |η*|_min_ of 14.4 Pa·s at 360 °C and Δ*T*_100_ of 320–380 °C, exhibiting good melt processability.

**Figure 6:**
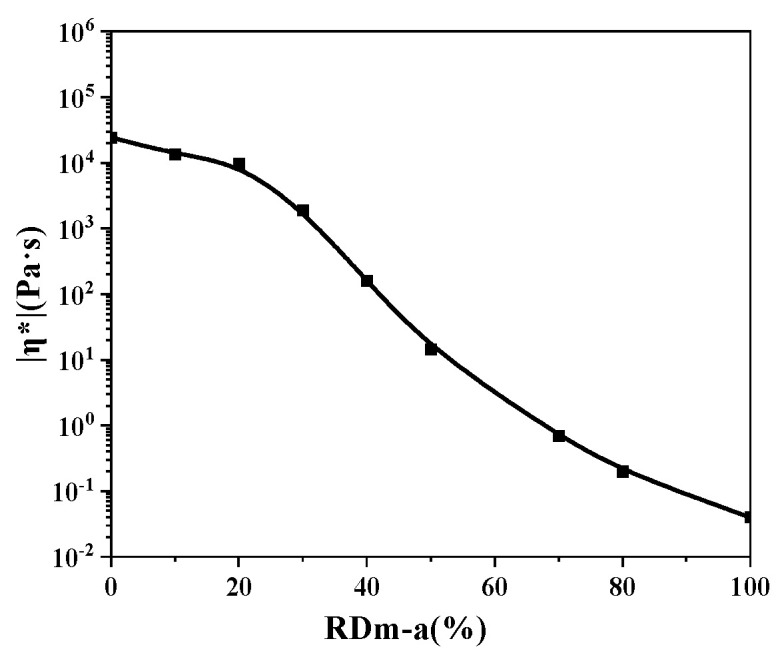
The minimum melt viscosity of the B-staged blend resins (TMR–50/RDm–a) with different RDm–a weight loadings.

Figure 6 depicts the effect of RDm–a weight loadings on the minimum melt viscosities of the B-staged blend resin (TMR–50/RDm–a). With an increase in the reactive diluent weight loading in the thermosetting blend resins, the minimum melt viscosity reduced significantly. When RDm–a weight loading is ≥50 wt.%, the B-staged blend resins showed minimum melt viscosity of lower than 100 Pa·s at <360 °C. For instance, the B-staged blend resin (TMR–50/RDm–a–5) exhibited the minimum melt viscosity of 14.4 Pa·s at 360 °C, and Δ*T*_10_ was measured at 320–380 °C, indicating a broad processing window (Figure 5b). The minimum melt viscosity of the B-staged blend resins decreased linearly with the increasing of RDm–a weight ratios of 20 % to 60 %, significantly improving the blend resin’s melt processability. The minimum melt viscosity of the blended resin (TMR–50/RDm–a-2) with 20% of RDm–a weight loading was measured at 1.0 × 10^4^ Pa·s at 365 °C, which was reduced to 10 Pa·s at 360 °C at 60% of RDm–a weight loading. When RDm–a loading was changed from 60% to 80%, the minimum melt viscosity decreased slightly from 10 Pa·s to 0.2 Pa·s. The TMR–50/RDm–a–5 with 50% of RDm–a weight loading showed a minimum melt viscosity of 14.4 Pa·s at 351 °C and Δ*T*_100_ of 320–380 °C, desirable for autoclave processing of carbon fiber composite for high temperature applications.

The results show that the molecular weight has a significant effects on the modifying results of diluent blends, the lower the molecular weight the more obvious the effect on the optimization of processing properties, and the method has strong applicability to resins with molecular weights between 1500 and 10,000.

### 3.3. Thermal Curing Kinetics of the B-Staged Blend Resins

**Figure 7:**
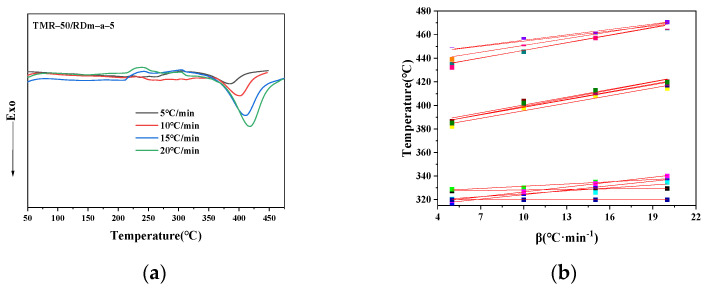
DSC curves (**a**) of the B-staged blend resin at different heating rates and the plot of the characteristic temperatures with heating rates (**b**).

In order to explore optimal curing conditions and curing kinetics of the thermosetting blend resins, non-isothermal DSC tests were performed at different heating rates of 5, 10, 15 and 20 °C /min (Figure 7a and Table 4). The experimental results indicated that the characteristic temperatures including initial curing (*T*_i_), exothermic peak (*T*_p_), and terminal curing (*T*_t_) all shifted to higher temperature area as the heating rate increased. For instance, when the heating rate increased from 5 °C/min to 20 °C/min, the *T*_p_ of TMR–50/RDm–a–5 increased from 384.9 °C to 418.2 °C, and *T*_t_ was increased from 447.2 °C to 470.5 °C, respectively. This could be interpreted by the enhanced thermal effect and larger temperature difference at higher heating rates. The characteristic temperatures (*T*_i_, *T*_p_ and *T*_t_) of the B-staged blend resin are plotted as the function of heating rates (Figure 7b). It can be seen that all of the characteristic temperatures increased linearly with increasing heating rates.

The characteristic temperatures at a heating rate of 0 °C /min were obtained by extrapolation. These parameters are very important to optimize the thermal curing process of the thermosetting blend resins, for the reason that a theoretical starting curing temperature is provided, which provides a theoretical basis for the setting of process procedures [41]. The typical temperatures of the blend resins increased to some extent as Clac’d M_w_ increased, implying that the thermal curing process could not be affected significantly by the molecular weight of the thermosetting polyimide resins.

The chain flexibility of the blended resin increased with expanding of polymer chain length. Therefore, the reactants need to overcome a lower energy barrier, resulting in increased activation energy for the thermal curing reaction. The reaction rate is proportional to the number of active sites available for chemical reactions. The blend resins with lower Clac’d M_w_ have much higher concentrated phenylethynyl groups, resulting in a more intensive collision between phenylethynyl groups and a higher frequency factor. The frequency factor of the blend resins showed obvious decreases with the increase of Clac’d M_w_.

If the thermal curing of the material follows the *n*th-order kinetics, the rate of curing reaction *d*α*/dt* is usually expressed as the following equation:

(1) $$\frac{d\alpha }{dt}=\mathrm{Aexp}\left(-\frac{E}{\mathrm{RT}}\right)\;(1\;-{\;\alpha )}^{n}$$

where *α* is the degree of cure, (1−*α*)^n^ is a function of the fractional extent of conversion, *t* is the reaction time, d*α*/dt is the rate of conversion.

Based on the results described above, the curing behavior of the blend resins (TMR–50/RDm–a–5) can be expressed by the following equation:

(2) $$\frac{d\alpha }{dd}=5{.33\;\times \;10}^{6}\;\exp\left(-\frac{147}{8.314\;\times \;T}\right){(1\;-\;\alpha )}^{0.93}$$

**Scheme 2:**
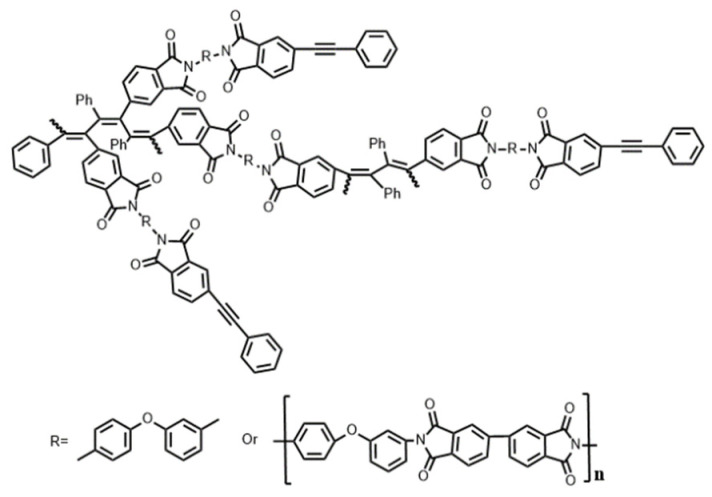
Proposed curing reactions of the thermosetting blend resins.

The results indicated that the thermosetting blend resins followed the first-order kinetics of phenylethynyl-terminated resins [42]. A two-staged kinetic/diffusion model could be used to explain the curing process of the thermosetting blend resins, which possess more dynamic endcaps and more rigid molecular chains [39]. The dominating reactions during thermal cure are ethynyl to ethynyl addition. A proposed curing reaction in the thermosetting blend resins is shown in Scheme 2. Furthermore, the blended resin cures were prepared according to the curing process obtained from the kinetic guidance of the resin curing reaction.

### 3.4. Thermal Stabilities of the Thermally Cured Blend Resins

**Figure 8:**
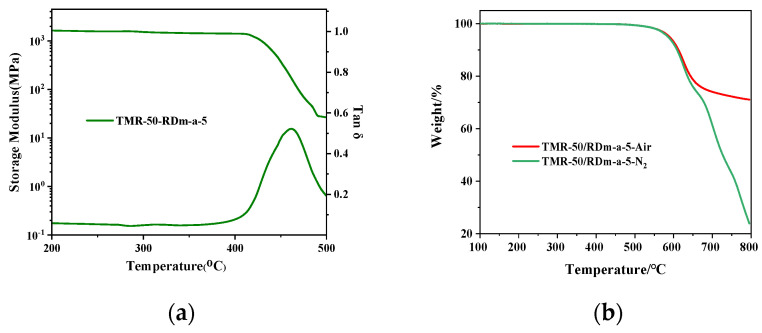
DMA (**a**) and TGA (**b**) of the thermally cured blend resins at 380 °C/2 h.

Figure 8a depicts the DMA curves of a representative thermosetting blend resin (TMR–50/RDm–a–5), which are the storage modulus and loss tangent (tan δ) as a function of temperature. Table 5 compares the characteristic temperatures including the onset temperature of storage modulus curves (*T*_onset_), the glass transition temperatures (*T*_g_). The *T*_onset_ values defined as the onset temperature of the storage modulus curve were measured in the range of 413–434 °C, and the *T*_g_ values defined as the peak temperature of tan δ curves were recorded in the range of 454–486 °C, which was decreased gradually with the increasing of Calc’d M_w_, probably due to the lowering in the crosslinked density of the blend resins. The lower crosslinking density could result in an increase in the free volume of the cured blend resins [43]. The thermally cured blend resin (TMR–50/RDm–a–5) showed the *T*_onset_ of 423 °C and *T*_g_ of 462 °C, respectively, suitable for 370 °C carbon fiber composite applications.

**Figure 9:**
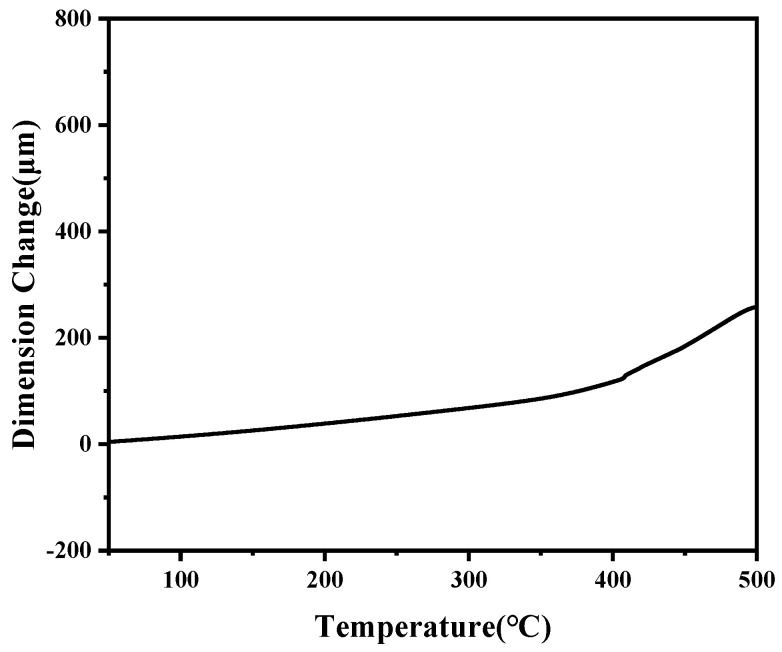
TMA of the thermally cured blend resins.

Figure 8b depicts the TGA curves of a representative thermosetting blend resin (TMR–50/RDm–a–5) at a heating rate of 10 °C/min in N_2_ and air, respectively. The initial decomposition temperature (*T*_d_), temperatures at 5% and 10% weight loss (*T*_d5_ and *T*_d10_) of the thermally cured blend resins are summarized in Table 5. The thermally cured blend resins with different Calc’d M_w_ showed excellent thermal and thermo-oxidatively stabilities. The *T*_d_ in N_2_ and in air were measured in the range of 588.5 to 597.7 °C and 585.0 to 594.2 °C, respectively. And the *T*_d10_ in N_2_ and in air were measured in the range of 608.8 to 618.1 °C and 605.3 to 614.6 °C, respectively. The high char yield of 75% and 39% at 800 °C in nitrogen and air were also measured, probably attributed to the higher rigidity of cross-linking network via the growing crosslinking density. Meanwhile, it is obvious that the increasing molecular weight of the matrix resins has no apparent impact on the thermal performance of the blends system. No remarkable thermal decomposition was detected until the temperature was scanned to 530 °C, demonstrating that all of the thermally cured blend resins possess outstanding heat resistance. The TMA curve showed no critical thermal expansion under 400 °C. The linear coefficient of thermal expansion (CTE) of TMR–50/RDm–a–5 is 5.6 × 10^−5^ °C^−1^ in the range of 40–250 °C (Figure 9). It was found that the blended cured resins exhibited excellent thermal stability.

### 3.5. Mechanical Properties of the Thermally Cured Blend Resins

**Figure 10:**
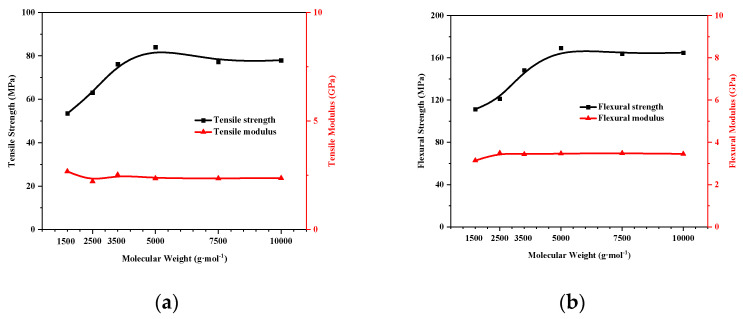
Mechanical properties of the thermally-cured blend resins (**a**) Flexural properties; (**b**) tensile properties.

Table 6 compares the flexural and tensile properties of the thermally cured blend resins at room temperature. With the increase of Calc’d M_w_ from 1500 to 5000 g/mol, the tensile strength of the thermally cured blend resins increased from 53.5 MPa to 84.0 MPa, and the flexural strength increased from 113.3 MPa to 169.2 MPa, respectively (Figure 10). This is mainly attributed to the polymer chain-extension by increasing the Calc’d M_w_. When Calc’d M_w_ is higher than 7500 g/mol, the tensile strength and flexural strength kept almost constant, without further increasing, indicating that the impact of polymer chain length on mechanical properties was not apparent. The elongation at breakage was also increased from 2.6% to 4.1% with increasing of Calc’d M_w_ from 1500 g/mol to 5000 g/mol, and then become almost constant with a further increase of Calc’d M_w_ from 7500 to 10,000 g/mol. The tensile and flexural modulus did not change obviously with the changing of Calc’d M_w_. Overall, TMR–50/RDm–a–5 showed the best combined mechanical properties with the tensile strength of 84.0 MPa, tensile modulus of 2.4 GPa, elongation at breakage of 4.1%, the flexural strength of 169.2 MPa, and flexural modulus of 3.5 GPa, respectively. This result also agreed with the reaction mechanism of the PEPA which was mainly by chain extension rather than cyclization [44,45]. So we next compounded this system with T700 carbon fiber to prepare polyimide based carbon fiber composites.

### 3.6. Mechanical Properties of Polyimide/Carbon Fiber Composites

Table 7 summarizes the mechanical properties of the polyimide/carbon fiber composite laminate (TMR–50/RDm–a–5/T700). The volume fraction of carbon fiber in the composite was measured at 62 ± 1. The flexural strength of the composite laminate was measured at 1552 MPa at 25 °C and 690 MPa at 370 °C, respectively. The flexural modulus at 119 GPa at 25 °C and 117 GPa at 370 °C, respectively, and the interlaminate shear strength at 95.2 MPa at 25 °C and 37.4 MPa at 370 °C, respectively. After post-cured at 400 °C/1 h + 420 °C/1 h, the composite laminate showed significantly improved mechanical properties. The retention of flexural strength at 370 °C was measured at 57.4% and the retention of interlaminar shear strength at 370 °C was 48.2%, much better than the as prepared composite. The flexural modulus increased about 14.3–15.3% after post-curing at 420 °C/1 h, and the retention of flexural modulus is as high as 99.3%, indicating that no obvious modulus loss at high temperature was detected.

## 4. Conclusions

The thermosetting poly(amic ester) matrix resin solution was blended with the reactive diluent (RDm) solution at different weight ratios to produce a series of A-staged thermosetting blend resin (TMR/RDm) solutions, which showed significantly improved melt processability and excellent thermal stabilities, being desirable as matrix resin of carbon fiber composite for 370 °C applications. The B-staged blend resins thermally treated at 200 °C/1 h showed low melt viscosities and a wider processing window. The minimum viscosities of ≤50 Pa·s was measured at ≤368 °C and the temperature scale at melt viscosities of ≤100 Pa·s were measured at 310–390 °C, respectively. The typical thermally cured neat resins at 380 °C/2 h showed a great combination of mechanical and thermal properties, including tensile strength of 84.0 MPa, elongation at breakage of 4.1%, and glass transition temperature (T_g_) of 423 °C, successively. The carbon fiber reinforced polyimide composite has been processed by autoclave technique, which exhibited excellent mechanical properties both at room temperature and at 370 °C. Based on these results, we believe that the excellent properties of the blend oligomers suggest a promising potential for application to be served at high temperatures in the aerospace and military industry to prepare structural parts with complex structures.

## Figures and Tables

**Table 1:** Compositions of the thermosetting blend matrix resins.

Blended Matrix Resins	Matrix Resin (50%)	Reactive Dilutes (50%)
TMR-15/RDm–a–5	TMR–15	RDm–a
TMR-25/RDm–a–5	TMR–25	
TMR-35/RDm–a–5	TMR–35	
TMR–50/RDm–a–5	TMR–50	
TMR-75/RDm–a–5	TMR–75	
TMR-100/RDm–a–5	TMR–100	
TMR–50/RDm–b–5	TMR–50	RDm–b
TMR–50/RDm–c–5	TMR–50	RDm–c
TMR–50/RDm–d–5	TMR–50	RDm–d

**Table 2:** The minimum melt viscosities of reactive diluents and the B-staged blend resins TMR–50/RDm–*x*–5 at a weight ratio of 50/50.

Reactive Diluents (RDm–*x*)			TMR–50/RDm–*x*–5	
Calc’d M_w_	|η*|_min_ (Pa·s/°C)	Δ*T_10_* (°C)	|η*|_min_ (Pa·s)	Δ*T*_100_ (°C)
660 (RDm–a)	0.1 (311 °C)	275–375 °C	14.4 (360 °C)	320–380 °C
1120 (RDm–b)	0.4 (347 °C)	250–375 °C	3.8 (358 °C)	293–380 °C
1580 (RDm–c)	1.5 (355 °C)	300–375 °C	39.8 (363 °C)	335–382 °C
2040 (RDm–d)	4.7 (370 °C)	340–380 °C	86.1 (360 °C)	363–375 °C

*x* = different serial number(a, b, c, d); |η*|_min_: Minimum melt viscosity; Δ*T_10_*: Temperature scale at |η*| < 10 Pa·s; Δ*T*_100_: Temperature scale at |η*|< 100 Pa·s.

**Table 3:** The minimum melt viscosities of the B-staged blend resins TMR/RDm–a–5 with different Calc’d M_w_ of TMR.

Samples	|η*|_min_ (Pa·s/°C)	Δ*T*_100_ (°C)
TMR–15/RDm–a–5	1.70 (343 °C)	317–383 °C
TMR–25/RDm–a–5	3.45 (358 °C)	323–388 °C
TMR–35/RDm–a–5	4.49 (363 °C)	316–380 °C
TMR–50/RDm–a–5	14.4 (360 °C)	320–380 °C
TMR–75/RDm–a–5	34.3 (368 °C)	342–379 °C
TMR–100/RDm–a–5	40.6 (366 °C)	343–378 °C

|η*|_min_: Minimum melt viscosity; Δ*T*_100_: Temperature scale at |η*| < 100 Pa·s.

**Table 4:** DSC data of the B-staged blend resin at different heating rates.

Sample	β (°C·min^−1^)	*T*_i_ (°C)	*T*_p_ (°C)	*T*_t_ (°C)	∆*T* (°C)
TMR–50/RDm–a–5	5	317.0	384.9	447.2	130.2
	10	355.0	402.0	456.2	101.2
	15	336.5	410.8	472.5	136.0
	20	329.0	418.2	470.5	141.5

*T*_i_: initial curing temperature; *T*_p_: exothermic peak temperatue; *T*_t_: terminal curing temperature.

**Table 5:** Thermal properties of the thermally-cured blend neat resins.

Samples	DMA		TGA in N_2_			TGA in Air		
	*T_o_*^1^ (°C)	*T*_g_^2^ (°C)	*T*_d_ (°C)	*T*_d5_ (°C)	*T*_d10_ (°C)	*T*_d_ (°C)	*T*_d5_ (°C)	*T*_d10_ (°C)
TMR–15/RDm–a–5	434	486	597.7	594.8	618.1	594.2	591.3	614.6
TMR–25/RDm–a–5	424	478	595.6	591.4	614.5	592.1	587.9	611.0
TMR–35/RDm–a–5	419	467	593.2	590.6	613.3	589.7	587.1	609.8
TMR–50/RDm–a–5	423	462	591.5	591.2	612.9	588.0	587.7	609.4
TMR–75/RDm–a–5	414	458	589.9	586.9	610.4	586.4	583.4	606.9
TMR–100/RDm–a–5	413	454	588.5	585.8	608.8	585.0	582.3	605.3

*T*_o_^1^: the onset temperature of storage modulus curve; *T*_g_^2^: the peak temperature of the tan δ curve.

**Table 6:** Mechanical properties of the cured resins.

Samples	Tensile Properties			Flexural Properties	
	Strength (MPa)	Modulus (GPa)	Elongation (%)	Strength (MPa)	Modulus (GPa)
TMR–15/RDm–a–5	53.5 (±3.10)	2.7 (±0.08)	2.6 (±0.48)	111.3 (±5.00)	3.1 (±0.09)
TMR–25/RDm–a–5	63.0 (±2.55)	2.2 (±0.09)	3.5 (±0.40)	121.3 (±5.60)	3.5 (±0.08)
TMR–35/RDm–a–5	76.2 (±4.31)	2.5 (±0.08)	3.2 (±0.32)	148.2 (±5.33)	3.4 (±0.08)
TMR–50/RDm–a–5	84.0 (±4.36)	2.4 (±0.10)	4.1 (±0.50)	169.2 (±4.58)	3.5 (±0.10)
TMR–75/RDm–a–5	77.2 (±3.18)	2.4 (±0.08)	3.9 (±0.44)	163.9 (±4.90)	3.5 (±0.10)
TMR–100/RDm–a–5	77.9 (±2.99)	2.4 (±0.10)	3.8 (±0.41)	164.8 (±2.70)	3.5 (±0.09)

**Table 7:** Mechanical properties of the polyimide/carbon fiber composites.

Property	Test Temperature (°C)	Composite As-Prepared (380 °C/2 h)	Composite after Post-Cured (420 °C/1 h)
Volume fraction of carbon fiber	--	62 (±1)	62(±1)
Flexural strength (MPa)	25	1552 (±48.4)	1663 (±34.9)
	370	690 (±15.1)	955 (±23.5)
Flexural modulus (GPa)	25	119 (±4.2)	136 (±4.0)
	370	117 (±1.1)	135 (±5.9)
Interlaminar shear strength (MPa)	25	95.2 (±2.4)	85.3 (±3.8)
	370	37.4 (±2.4)	41.3 (±1.0)

## Data Availability

In this No new data were created or analyzed in this study. Data sharing is not applicable to this article.
